# Tweet Classification Toward Twitter-Based Disease Surveillance: New Data, Methods, and Evaluations

**DOI:** 10.2196/12783

**Published:** 2019-02-20

**Authors:** Shoko Wakamiya, Mizuki Morita, Yoshinobu Kano, Tomoko Ohkuma, Eiji Aramaki

**Affiliations:** 1 Institute for Research Initiatives Nara Institute of Science and Technology Ikoma Japan; 2 Graduate School of Science and Technology Nara Institute of Science and Technology Ikoma Japan; 3 Data Science Center Nara Institute of Science and Technology Ikoma Japan; 4 Okayama University Okayama Japan; 5 Shizuoka University Hamamatsu Japan; 6 Fuji Xerox Co., Ltd. Yokohama Japan

**Keywords:** text mining, social media, machine learning, natural language processing, artificial intelligence, surveillance, infodemiology, infoveillance

## Abstract

**Background:**

The amount of medical and clinical-related information on the Web is increasing. Among the different types of information available, social media–based data obtained directly from people are particularly valuable and are attracting significant attention. To encourage medical natural language processing (NLP) research exploiting social media data, the 13th NII Testbeds and Community for Information access Research (NTCIR-13) Medical natural language processing for Web document (MedWeb) provides pseudo-Twitter messages in a cross-language and multi-label corpus, covering 3 languages (Japanese, English, and Chinese) and annotated with 8 symptom labels (such as cold, fever, and flu). Then, participants classify each tweet into 1 of the 2 categories: those containing a patient’s symptom and those that do not.

**Objective:**

This study aimed to present the results of groups participating in a Japanese subtask, English subtask, and Chinese subtask along with discussions, to clarify the issues that need to be resolved in the field of medical NLP.

**Methods:**

In summary, 8 groups (19 systems) participated in the Japanese subtask, 4 groups (12 systems) participated in the English subtask, and 2 groups (6 systems) participated in the Chinese subtask. In total, 2 baseline systems were constructed for each subtask. The performance of the participant and baseline systems was assessed using the exact match accuracy, F-measure based on precision and recall, and Hamming loss.

**Results:**

The best system achieved exactly 0.880 match accuracy, 0.920 F-measure, and 0.019 Hamming loss. The averages of match accuracy, F-measure, and Hamming loss for the Japanese subtask were 0.720, 0.820, and 0.051; those for the English subtask were 0.770, 0.850, and 0.037; and those for the Chinese subtask were 0.810, 0.880, and 0.032, respectively.

**Conclusions:**

This paper presented and discussed the performance of systems participating in the NTCIR-13 MedWeb task. As the MedWeb task settings can be formalized as the factualization of text, the achievement of this task could be directly applied to practical clinical applications.

## Introduction

Medical reports using electronic media are now replacing those of paper media [1,2]. As a result, the importance of natural language processing (NLP) techniques in various medical fields has increased significantly. Currently, the development of practical tools to assist precise and timely medical decisions has been encouraged.

To contribute to the progress of information retrieval research, a series of *shared tasks* (or contests, competitions, challenge evaluations, and critical assessments) is being used. Thus far, several shared tasks related to medical or health care have already been organized and provided datasets for various NLP tasks. These include the Informatics for Integrating Biology and the Bedside (i2b2) tasks [3], the Text Retrieval Conference (TREC) Medical Records track [4], TREC Clinical Decision Support or Precision Medicine tracks [5-9], the Cross-Language Evaluation Forum for European Languages (CLEF) eHealth [10], and NII Testbeds and Community for Information access Research (NTCIR) Medical tasks and Medical Natural Language Processing (MedNLP) workshops [11-16]. Generally, these shared tasks provide clinical records.

On the other hand, with the widespread use of the internet, considerable material concerning medical or health care has been shared on the Web, and several Web mining techniques for utilizing the material have been developed. One of the most popular medical applications of Web mining is flu surveillance [17-27]. Although most previous studies have relied on shallow textual clues in messages, such as the number of occurrences of specific keywords (eg, *flu* or *influenza*), such simple approaches have difficulty coping with the volume of noisy messages. Typical examples of noisy tweets on Twitter are those that simply express concern or awareness about flu (such as “Starting to get worried about swine flu”). To increase their accuracy, one of the most reasonable approaches employs a binary classifier to filter out noisy messages.

Given this situation, the NTCIR-13 [28] Medical Natural Language Processing for Web Document (MedWeb) task [29,30] is designed for obtaining health-related information by exploiting data on the Web, focusing on social media sites such as Twitter [31] and Facebook [32]. Specifically, we propose a generalized task setting that determines whether a message is written about a patient affected by a specific symptom for public health surveillance, referring to the following 2 characteristics:

1. Multi-label: This task handles not only a single symptom (such as influenza) but also multiple symptoms such as cold, cough or sore throat, diarrhea or stomach ache, fever, hay fever, headache, and runny nose. As a single message can contain multiple symptoms, this is a multi-labeling task.
2. Cross-language: In contrast to the previous shared tasks, this task covers multiple languages, such as Japanese, English, and Chinese. To build parallel corpora, we translated the original Japanese messages to English and Chinese.

In the NTCIR-13 MedWeb, we distributed each corpus to the participants [33-41], of whom 9 groups (7 academia groups, an industry group, and a joint group) submitted results (37 systems). Specifically, 8 groups (19 systems) participated in the Japanese subtask, 4 groups (12 systems) participated in the English subtask, and 2 groups (6 systems) participated in the Chinese subtask (see Multimedia Appendix 1). This report presents the results of these groups, along with discussions, to clarify the issues that need to be resolved in the field of medical NLP.

## Methods

### Materials

#### Data

The MedWeb task uses a collection of tweets that include at least one keyword of target diseases or symptoms (for brevity, we refer to these simply as *symptoms* hereafter). We set 8 symptoms, including cold, cough or sore throat (which we refer to as *cough*), diarrhea or stomachache (*diarrhea*), fever, hay fever, headache, influenza (*flu*), and runny nose.

Owing to the Twitter Developer Policy on data redistribution [42], the tweet data crawled using the application programming interface [43] are not publicly available. Therefore, our data consist of pseudotweets created by a crowdsourcing service.

To obtain the pseudotweets, we first collected Japanese tweets related to each symptom from Twitter. Then, we classified these tweets as positive or negative based on the previous study [19]. Next, we extracted keyword sets that appeared frequently in the positive and negative tweets of the symptom by calculating term frequency and inverse document frequency. We call these keywords *seed words*.

We then had a group of people create pseudotweets consisting of 100 to 140 characters, which included a symptom and at least one of the seed words of the symptom. Each person created 32 pseudotweets (2 tweets × 2 keyword sets [positive and negative] × 8 symptoms). As a result, 80 people were able to generate 2560 Japanese pseudotweets.

In the last step, we had the Japanese pseudotweets translated into English and Chinese by relevant first-language practitioners. Therefore, we also had 2560 pseudotweets in both English and Chinese. The corpora are available in a previous paper [44]. Textbox 1 shows samples of each set of pseudotweets, whose ratios of positive labels are presented in Table 1. This table shows the ratio of positive labels out of each symptom’s 320 pseudotweets and the number of positive labels out of all symptoms’ 2560 pseudotweets. Common symptoms such as a runny nose, fever, headache, and cold tend to appear with the other symptoms. Then, the number of tweets of the flu labeled as *p* (positive) is relatively less than the others, indicating that the flu is likely to be a topic even if people did not suffer from flu. On the other hand, tweets concerning several symptoms such as a cough, headache, runny nose, and diarrhea are described in many cases when people suffered from them.

**Samples of pseudotweets of the 8 symptoms. Note that English messages and Chinese messages were translated from Japanese messages.**

1. Cold
  - 風邪を引くと全身がだるくなる
  - The cold makes my whole body weak.
  - 一感冒就浑身酸软无力。
2. Cough
  - あかん。咳込みすぎて頭まで痛くなってきた
  - This is not good. I coughed too much and I got a headache from it.
  - 糟了。咳得太厉害，头都疼起来了。
3. Diarrhea
  - 下痢ひどすぎで笑うわ
  - I gotta laugh. My diarrhea is so bad.
  - 腹泻过于严重，很搞笑。
4. Fever
  - 熱が出なくてもリンパが腫れることがよくある。
  - It’s not unusual for lymph nodes to get swollen, even when there’s no fever.
  - 很多时候就算不发热淋巴也肿。
5. Hay fever
  - 花粉症の症状が出てきたのは久し振りだ。
  - It’s been a while since I’ve had allergy symptoms.
  - 好久没有出现花粉症的症状了。
6. Headache
  - 頭痛がやばいから帰宅して寝るー
  - My headache is killing me, so I’m going to go home and sleep.
  - 因为头疼得厉害，我回家睡觉了。
7. Flu
  - インフルエンザのワクチン打ちに行ってきた。
  - I went to get vaccinated for the flu.
  - 去打了流感的疫苗。
8. Runny nose
  - 鼻づまりで今日は休むわー
  - I’m not going today, because my stuffy nose is killing me.
  - 因为鼻塞，今天休息吧！

**Table 1:** Ratio of positive labels.

Symptom	Ratio of number of positive tweets to the number of each symptom’s tweets (N=320 tweets)	Ratio of number of positive tweets to the total number of all symptoms’ tweets (N=2560 tweets)
Cold, n (%)	220 (0.6875)	355 (0.1387)
Cough, n (%)	295 (0.9219)	306 (0.1195)
Diarrhea, n (%)	230 (0.7188)	246 (0.0961)
Fever, n (%)	220 (0.6875)	438 (0.1711)
Hay fever, n (%)	208 (0.6500)	209 (0.0816)
Headache, n (%)	260 (0.8125)	328 (0.1281)
Flu, n (%)	128 (0.4000)	130 (0.0508)
Runny nose, n (%)	257 (0.8031)	499 (0.1949)

**Table 2:** Samples of the training data corpus for the English subtask.

Tweet ID	Message	s_1_^a^	s_2_	s_3_	s_4_	s_5_	s_6_	s_7_	s_8_
1en^b^	The cold makes my whole body weak.	p^c^	n^d^	n	n	n	n	n	n
2en	It’s been a while since I’ve had allergy symptoms.	n	n	n	n	p	n	n	p
3en	I’m so feverish and out of it because of my allergies. I’m so sleepy.	n	n	n	p	p	n	n	p
4en	I took some medicine for my runny nose, but it won’t stop.	n	n	n	n	n	n	n	p
5en	I had a bad case of diarrhea when I traveled to Nepal.	n	n	n	n	n	n	n	n
6en	It takes a millennial wimp to call in sick just because they’re coughing. It’s always important to go to work, no matter what.	n	p	n	n	n	n	n	n
7en	I’m not going today, because my stuffy nose is killing me.	n	n	n	n	n	n	n	p
8en	I never thought I would have allergies.	n	n	n	n	p	n	n	p
9en	I have a fever but I don’t think it’s the kind of cold that will make it to my stomach.	p	n	n	p	n	n	n	n
10en	My phlegm has blood in it and it’s really gross.	n	p	n	n	n	n	n	n

^a^s_1_, s_2_, s_3_, s_4_, s_5_, s_6_, s_7_, and s_8_ are IDs of the 8 symptoms (cold, cough, diarrhea, fever, hay fever, headache, flu, and runny nose).

^b^ID corresponds to the corpora of other languages (eg, the tweet of *1en* corresponds to the tweets of *1ja* and *1zh*).

^c^p indicates the positive label.

^d^n indicates the negative label.

#### Symptom Labeling

This section describes the criteria used for symptom labeling: basic criteria and symptom-specific criteria [45,46]. In this study, 2 annotators attached positive or negative labels of the 8 symptoms to tweets (Table 2).

##### Basic Criteria

The most basic criterion is that the labeling is examined from a clinical viewpoint, considering the medical importance of the information. Thus, nonclinical information should be disregarded. For example, older information (by several weeks) and nonsevere symptoms (a headache due to overdrinking) should be labeled as *n* (negative).

The following 3 criteria describe the basic principles:

1. Factuality: The Twitter user (or someone close to the user) should be affected by a certain disease or have a symptom of the disease. A tweet that includes only the name of a disease or a symptom as a topic is removed by labeling it as *n* (negative).
2. Tense (time): Older information, which is meaningless from the viewpoint of surveillance, should be discarded. Such information should also be labeled as *n* (negative). Here, we regard 24 hours as the standard condition. When the precise date and time are ambiguous, the general guideline is that information within 24 hours (eg, information related to the present day or previous day) is labeled as *p* (positive).
3. Location: The location of the disease should be specified as follows. If a Twitter user is affected, the information is labeled as *p* (positive) because the location of the user is the place of onset of the symptom. In cases where the user is not personally affected, the information is labeled as *p* (positive) if it is within the same vicinity (prefecture) as that of the user, and as *n* (negative) otherwise.

##### Symptom-Specific Criteria

There are several exceptions to the fundamental annotation principles. For example, a remark about a *headache* might not relate to that about a clinical disease (such as headache due to excessive drinking). When conducting disease surveillance, such statements should be regarded as noise. To deal with disease-specific phenomena, we build a guideline that addresses exceptions for each disease. For example, cases such as *excessive drinking*, *medication*, *pungently flavored food (including irritant),* *spiritual,* *motion sickness,* *morning,* and *menstrual pain* should be excluded for *headache.* The exceptions are summarized in Table 3.

### Task Settings

In the MedWeb task, we organized 3 subtasks: a Japanese subtask, an English subtask, and a Chinese subtask. The procedure of the MedWeb task is as follows:

*Step 1.* Training corpus distribution: The training data corpus and the annotation criteria were sent to the participant groups for development. The training data corpus comprises 1920 messages (75.00% of the whole corpus), with labels. Each message is labeled *p* (positive) or *n* (negative) for each of the 8 symptoms.

*Step 2.* Formal run result submission: After about a 3-month development period, the test data corpus was sent to each participant group. The test data corpus consists of 640 messages (25.0% of the whole corpus), without labels. Then, the participant groups developed their systems (Table 4) and submitted their annotated results within 2 weeks. Multiple results with up to 3 systems were allowed to be submitted.

*Step 3.* Evaluation result release: After a 1-month evaluation period, the evaluation results and annotated test data were sent to each participant group.

### Systems

#### Baseline Systems

As a baseline, 2 systems were constructed using a support vector machine (SVM) based on unigram and bigram features. For feature representation, the bag-of-words model was used in each system. A tweet message was segmented using MeCab, created by Kudo et al [47] for Japanese messages, natural language toolkit (NLTK) TweetTokenizer, created by Bird [48,49] for English messages, and jieba, created by Junyi [50] for Chinese messages. The 2 systems had a linear kernel, and the parameter for regularization, *C*, was set to 1. The baseline systems were implemented using scikit-learn (sklearn) [51,52].

#### Participating Systems

In all, 37 systems (of 9 groups) participated and had their results submitted in the MedWeb. Of these, 19 systems (of 8 groups) submitted results for the Japanese subtask, 12 systems (of 4 groups) for the English subtask, and 6 systems (of 2 groups) for the Chinese subtask. The participating systems for the Japanese, English, and Chinese subtasks are summarized in Table 4.

**Table 3:** Exceptions for symptom labels.

Symptom	Expressions with suspicion	Just a symptom word	Exceptions	
			Regarded as symptom	Not regarded as symptom
Cold	Accept	Accept	—^a^	—
Cough	Accept	Accept	Alcohol drinking and pungently flavored food	—
Diarrhea	Accept	Accept	Overeating, indigestion, alcohol drinking, medication, and pungently flavored food	—
Fever	Accept	Only *slight fever*	Hay fever and side effect due to any injection	—
Hay fever	Accept	Accept	—	—
Headache	Accept	Accept	—	Due to a sense of sight or smell
Flu	Not accept	Not accept	—	—
Runny nose	Accept	Not accept	Hay fever	Change in temperature

^a^Indicates there are no exceptions.

**Table 4:** Participating systems in subtasks. A total of 19 participating systems and 2 baseline systems are constructed for the Japanese subtask, 12 participating systems and 2 baseline systems are constructed for the English subtask, and 6 participating systems and 2 baseline systems are constructed for the Chinese subtask.

System ID	Models or methods	Language resources
AITOK-ja [33]	Keyword-based, logistic regression, and SVM^a,b^	—^c^
AKBL-ja and AKBL-en [34]	SVM and Fisher exact test	Patient symptom feature word dictionary and Disease-X feature words dict1 and dict2
DrG-ja [35]	Random forest	—
KIS-ja [36]	Rule-based and SVM	—
NAIST-ja, NAIST-en, and NAIST-zh [37]	Ensembles of hierarchical attention network and deep character-level convolutional neural network with loss functions (negative loss function, hinge, and hinge squared)	—
NIL-ja [38]	Rule-based	—
NTTMU-ja [39]	Principle-based approach	Manually constructed knowledge for capturing tweets that conveyed flu-related information, using common sense and ICD-10^d^
NTTMU-en [39]	SVM and recurrent neural network	Manually constructed knowledge for capturing tweets that conveyed flu-related information, using common sense and ICD-10
TUA1-zh [40]	Logistic regression, SVM, and logistic regression with semantic information	Updated training samples using active learning unlabeled posts downloaded with the symptom names in Chinese
UE-ja [41]	Rule-based and random forest	Custom dictionary consisting of nouns selected from the dry-run dataset and heuristics
UE-en [41]	Rule-based, random forests, and skip-gram neural network for word2vec	Custom dictionary consisting of nouns selected from the dry-run dataset and heuristics
Baseline	SVM (unigram and bigram)	—

^a^SVM: support vector machine.

^b^It indicates that the method was tested after the submission of the formal run, and thus, it was not included in the results.

^c^It indicates that any language resources were not used.

^d^ICD: International Codes for Diseases.

As for the Japanese subtask, most of the groups applied machine learning approaches, such as SVM (as in the baseline systems), random forests, and neural networks. Several groups constructed their own resources to enhance the original training corpus. Similarly, for the English subtask, most of the groups applied machine learning approaches, such as SVM, random forests, and neural networks. The Chinese subtask had 2 participating groups: one applied the same methods as the other subtasks and the other used logistic regression and SVM and updated the training data using active learning.

### Evaluation Metrics

The performance in the subtasks was assessed using the exact match accuracy, F-measure (beta=1) (*F1*) based on precision and recall, and Hamming loss [53].

The details of the metrics are as follows.

- Exact match accuracy: If *y’*^*(i)*^ indicates the predicted symptom label values of the *i*-th tweet and *y*^*(i)*^ is the corresponding true labels, then the fraction of correct predictions over the test data corpus (*N*=640) is calculated as follows: *accuracy(y, y’)=1/N* *∙∑*^*N*^_*i=1*_*1(* *y’*^*(i)*^*=y*^*(i)*^*),* where *1(.)* is the indicator function, which returns 1 if the entire set of predicted labels for a tweet strictly matches with the true set of labels.
- Precision (micro or macro): It is defined as the number of true positives (*T*_*p*_) over the sum of the number of true positive and false positives (*F*_*p*_): *precision=T*_*p*_*/(T*_*p*_*+F*_*p*_*)*.
- Recall (micro or macro): It is defined as the number of true positives (*T*_*p*_) over the sum of the number of true positive and false negatives (*F*_*n*_): *recall=T*_*p*_*/(T*_*p*_*+F*_*n*_*)*.
- F1 (micro or macro): The harmonic mean of precision and recall is calculated as follows: *F1=2∙precision∙recall/(precision+recall).*
- Hamming loss: It computes the average Hamming loss between two sets of labels. If *y’*^*(i)*^ is the predicted value for the *j*-th label of the *i*-th tweet, *y*^*(i)*^_*j*_ is the corresponding true value, *N* is the number of test data (*N*=640), and *L* is the number of labels (*L*=8), then the Hamming loss between the predicted and correct labels is calculated as follows: *L*_*Hamming*_*(y,y’)=1/N* *∙1/L∙∑*^*N*^_*i=1*_*∑*^*L*^_*j=1*_*1(y’*^*(i)*^_*j*_*≠y*^*(i)*^_*j*_*),* where *1(.)* is the indicator function. Note that lower scores are better.

Note that *micro* is to calculate metrics globally by counting all true positives, false negatives, and false positives: *F1micro=2∙precisionmicro∙recallmicro/(precisionmicro+recallmicro)*.

On the other hand, *macro* calculates the metrics for each symptom label and then determines their unweighted mean: *F1macro=1/L∙∑*^*L*^_*j=1*_*F1*_*j*_*.* Therefore, label imbalance is not taken into account.

### Ethics Statement

This study did not require the participants to be involved in any physical and/or mental intervention. As this research did not use personally identifiable information, it was exempted from the institutional review board approval in accordance with the Ethical Guidelines for Medical and Health Research Involving Human Subjects stipulated by the Japanese national government.

## Results

### Symptom Labeling Reliability

To show the reliability of symptom labeling to the corpus, the interannotation agreement ratios of the respective symptoms were measured (Table 5). The total interannotator agreement ratio (n=2) was 0.9851 (ie, 20,174 / [2560 × 8]).

### Performance of Baseline Systems

The performance of the baseline was measured using all evaluation metrics. Tables 6-8 show the results for the Japanese, English, and Chinese subtasks, respectively.

For the Japanese and Chinese subtasks, unigram SVM performed better than bigram SVM. On the other hand, bigram SVM outperformed unigram SVM in the English subtask. The highest average of exact match accuracy was 0.791 (English subtask) and the lowest was 0.756 (Japanese subtask).

### Performance of Participating Systems

The performance of the participating systems was also measured using all evaluation metrics. Tables 6-8 show the results for the Japanese, English, and Chinese subtasks, respectively, ordered by the exact match accuracy of the systems.

For the Japanese subtask, the best system, NAIST-ja-2, achieved 0.880 in exact match accuracy, 0.920 in F-measure, and 0.019 in Hamming loss, as shown in Table 6. The averages across the participating groups and the baseline systems were 0.720, 0.820, and 0.051, respectively. The rank order of the top 4 systems was the same in all measures. The systems of the AKBL and KIS groups were constructed using an SVM, as in the baseline systems. The AKBL group’s results indicated that their system was effective in terms of using additional language resources. The KIS group switched their methods between an SVM and a rule-based method, depending on the confidence factor.

For the English subtask, the best system, NAIST-en-2, achieved 0.880 in exact match accuracy, 0.920 in F-measure, and 0.019 in Hamming loss, as shown in Table 7. The system was constructed using the same method as that used in the Japanese subtask. The averages across the participating groups and the baseline systems were 0.770, 0.850, and 0.037, respectively.

For the Chinese subtask, the best system, NAIST-zh-2, achieved 0.880 in exact match accuracy, 0.920 in F-measure, and 0.019 in Hamming loss, as shown in Table 8. The system was constructed using the same method as that used in the Japanese and English subtasks. The averages across the participating groups and the baseline systems were 0.810, 0.880, and 0.032, respectively.

**Table 5:** Interannotator agreement ratio.

Symptom	Agreement ratio (number)
Cold	0.9945 (2546/2560)
Cough	0.9934 (2543/2560)
Diarrhea	0.9785 (2505/2560)
Fever	0.9922 (2540/2560)
Hay fever	0.9918 (2539/2560)
Headache	0.9773 (2502/2560)
Flu	0.9734 (2492/2560)
Runny nose	0.9793 (2507/2560)
Total	0.9851 (20,174/20,480)

**Table 6:** Performance in the Japanese subtask (19 participating systems and 2 baseline systems).

System ID^a^	Exact match^b^	F1		Precision		Recall		Hamming loss
		Micro	Macro	Micro	Macro	Micro	Macro	
NAIST-ja-2	0.880	0.920	0.906	0.899	0.887	0.941	0.925	0.019
NAIST-ja-3	0.878	0.919	0.904	0.899	0.885	0.940	0.924	0.019
NAIST-ja-1	0.877	0.918	0.904	0.899	0.887	0.938	0.921	0.020
AKBL-ja-3	0.805	0.872	0.859	0.896	0.883	0.849	0.839	0.029
UE-ja-1	0.805	0.865	0.855	0.831	0.819	0.903	0.902	0.033
KIS-ja-2	0.802	0.871	0.856	0.831	0.815	0.915	0.904	0.032
AKBL-ja-1	0.800	0.869	0.847	0.889	0.873	0.849	0.825	0.030
UE-ja-3	0.800	0.866	0.855	0.823	0.812	0.913	0.911	0.033
AKBL-ja-2	0.795	0.868	0.849	0.891	0.875	0.846	0.827	0.030
KIS-ja-3	0.784	0.855	0.831	0.840	0.816	0.871	0.850	0.034
SVM-unigram	0.761	0.849	0.835	0.843	0.828	0.854	0.842	0.036
KIS-ja-1	0.758	0.849	0.833	0.798	0.782	0.906	0.899	0.038
SVM-bigram	0.752	0.843	0.830	0.838	0.820	0.848	0.845	0.037
NTTMU-ja-1	0.738	0.835	0.829	0.770	0.761	0.913	0.921	0.042
UE-ja-2	0.706	0.815	0.803	0.696	0.702	0.983	0.984	0.052
NIL-ja-1	0.680	0.749	0.742	0.862	0.845	0.662	0.671	0.052
DrG-ja-1	0.653	0.777	0.774	0.825	0.808	0.734	0.779	0.049
NTTMU-ja-3	0.614	0.775	0.773	0.740	0.720	0.814	0.840	0.055
NTTMU-ja-2	0.597	0.770	0.753	0.741	0.706	0.801	0.813	0.056
AITOK-ja-2	0.503	0.706	0.696	0.726	0.738	0.687	0.767	0.067

^a^The system ID comprises the group ID (see Multimedia Appendix 1), the abbreviation of subtask (ja indicates Japanese subtask), and the system number from 1 to 3 since each group can submit three systems per subtask.

^b^The results are ordered by exact match accuracy.

**Table 7:** Performance in the English subtask (12 participating systems and 2 baseline systems).

System ID^a^	Exact match^b^	F1		Precision		Recall		Hamming loss
		Micro	Macro	Micro	Macro	Micro	Macro	
NAIST-en-2	0.880	0.920	0.906	0.899	0.887	0.941	0.925	0.019
NAIST-en-3	0.878	0.919	0.904	0.899	0.885	0.940	0.924	0.019
NAIST-en-1	0.877	0.918	0.904	0.899	0.887	0.938	0.921	0.020
SVM-bigram	0.800	0.866	0.856	0.865	0.849	0.868	0.865	0.031
UE-en-1	0.789	0.858	0.848	0.846	0.831	0.871	0.876	0.034
SVM-unigram	0.783	0.858	0.845	0.851	0.830	0.864	0.864	0.033
NTTMU-en-2	0.773	0.856	0.849	0.807	0.796	0.911	0.918	0.036
NTTMU-en-3	0.758	0.845	0.828	0.836	0.818	0.854	0.844	0.037
UE-en-2	0.745	0.821	0.809	0.861	0.838	0.786	0.800	0.040
UE-en-3	0.739	0.820	0.815	0.870	0.851	0.776	0.795	0.040
AKBL-en-2	0.734	0.819	0.799	0.832	0.808	0.806	0.793	0.042
AKBL-en-3	0.716	0.804	0.787	0.853	0.834	0.760	0.747	0.043
NTTMU-en-1	0.619	0.770	0.777	0.734	0.733	0.809	0.835	0.056
AKBL-en-1	0.613	0.772	0.755	0.656	0.649	0.936	0.945	0.065

^a^The system ID comprises the group ID (see Multimedia Appendix 1), the abbreviation of subtask (en indicates English subtask), and the system number from 1 to 3 since each group can submit three systems per subtask.

^b^The results are ordered by exact match accuracy.

**Table 8:** Performance in the Chinese subtask (6 participating systems and 2 baseline systems).

System ID^a^	Exact match^b^	F1		Precision		Recall		Hamming loss
		Micro	Macro	Micro	Macro	Micro	Macro	
NAIST-zh-2	0.880	0.920	0.906	0.899	0.887	0.941	0.925	0.019
NAIST-zh-3	0.878	0.919	0.904	0.899	0.885	0.940	0.924	0.019
NAIST-zh-1	0.877	0.918	0.904	0.899	0.887	0.938	0.921	0.020
TUA1-zh-3	0.786	0.860	0.844	0.772	0.760	0.970	0.971	0.037
SVM-unigram	0.780	0.858	0.843	0.831	0.815	0.888	0.883	0.034
TUA1-zh-1	0.773	0.853	0.838	0.766	0.753	0.963	0.965	0.039
SVM-bigram	0.767	0.850	0.835	0.824	0.806	0.878	0.876	0.036
TUA1-zh-2	0.719	0.824	0.809	0.712	0.710	0.978	0.982	0.049

^a^The system ID comprises the group ID (see Multimedia Appendix 1), the abbreviation of subtask (zh indicates Chinese subtask), and the system number from 1 to 3 since each group can submit 3 systems per subtask.

^b^ The results are ordered by exact match accuracy.

## Discussion

### Principal Findings

One of the most valuable findings was that we could determine the best strategy for disease surveillance. The best system of the NAIST group had 2 characteristics: (1) cross-language features and (2) ensemble of multiple machine learning methods.

#### Cross-Language Features

For each language, the NAIST system utilized features from the other 2 languages. English and Chinese sentences were translated from a Japanese sentence, indicating that these 3 sentences shared the same symptom label set. Only the NAIST system focused on the property of this task’s corpus and improved the accuracy from 0.767 to 0.823 in exact match.

#### Ensemble Methods

The NAIST system also utilized an ensemble method, which combines multiple methods to boost the classification accuracy. Although weak machine learning algorithms tend to be generally preferred to make an ensemble, the NAIST group created an ensemble consisting of strong machine learning methods: a hierarchical attention network and a deep convolutional neural network (CNN). The combination of methods varied the exact match accuracy of 0.836 at the minimum to 0.880 at the maximum. In the near future, a technique to find a better combination needs to be developed.

Out of the 2 features, the cross-language feature is the unique feature of this task. Even if we discounted the cross-language feature, the NAIST ensemble method exhibited the best performance. As the multi-label classification is known as a complex task, the performance of straightforward approaches relying only on 1 method was relatively lower than that of the NAIST system.

Note that previous NTCIR medical tasks and MedNLP workshops [13-15] have shown that a rule-based approach is still competitive with machine learning approaches. One of the reasons for this was the small size of the corpus they used. Although the corpus size was also limited in this task, this result showed the advantage of complex machine learning, indicating the advancement of machine learning techniques.

### Subtask-Based Comparison

The MedWeb task provided a cross-language corpus. Although this is another characteristic of this task, only 1 group (NAIST) challenged all subtasks, which was lesser than our expectation. The Japanese subtask had the highest participation (19 systems from 8 groups), whereas the Chinese subtask had the lowest participation (6 systems from only 2 groups), which was also lower than our expectation.

**Figure 1:**
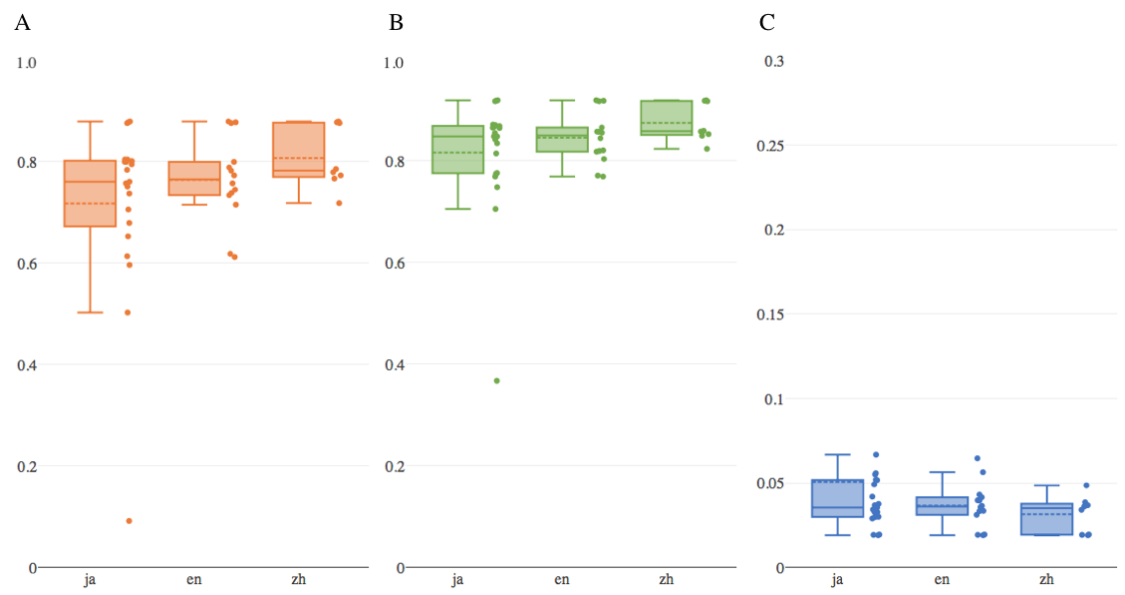
Statistical summary of the performance of 3 evaluation metrics (A: Exact math accuracy, B: F1-micro, and C: Hamming loss) in each of the subtasks (ja: Japanese, en: English, and zh: Chinese). Note that higher scores are better in exact match accuracy and F1-micro, whereas lower scores are better in hamming loss. The bottom and top of a box are the first and third quartiles, the band inside the box is the median, and the dotted band inside the box is the mean. Dots on the right side of the box represent the distribution of values of participating systems.

The performance varied depending on the subtasks. Figure 1 shows the distribution of the 3 metric scores of the systems in each subtask. For the Japanese subtask, the performance varied widely, relative to that of the other subtasks. Although the Chinese subtask had the lowest participation, their performance was relatively high. The 4 groups that participated in the Japanese subtask also challenged the English subtask, with better results, on average, in the English subtask. This indicates that the difficulty of classification in increasing order is Chinese, English, and Japanese. This is a surprising result because most of the groups came from Japan and must have been familiar with the Japanese NLP.

This indicates that the Chinese language has less ambiguity in clinical factuality analyses. Another possibility is that the process we used to generate the corpora had a language bias. For example, the translations from Japanese to English and Chinese might have reduced the ambiguity of the language in each case. To test for language bias, experiments based on different directions of translation are necessary. This is left for future work. Note that the baseline systems performed the best in the English subtask, indicating that the standard settings for SVM are effective in terms of classifying English tweets.

### Limitations

The corpora provided by the MedWeb task have the following limitations. The first is the generating process of the corpora. For example, our pseudotweets do not include several tweet-specific features such as reply, retweet, hashtag, and URL. In addition, the translation process might bias the results. Although we asked translators to translate Japanese short messages without following standard English or Chinese as they could, some of them would be more formal than tweets.

Another limitation is the size of each corpus (1920 messages are used as training data and 640 messages are used as test data). Regardless of these limitations, we believe that this is a valuable attempt to generate and share a cross-language corpus consisting of multi-label pseudotweets.

Although our corpus has some limitations, we still believe it is helpful as a benchmark for tweet-based applications, because it is freely available and covers multiple languages.

### Comparison With Prior Work

Currently, several shared tasks related to medical or health care have already been held. In the United States, the i2b2 tasks [3] were organized by the National Institute of Health [54] to enhance the ability of NLP tools to extract fine-grained information from clinical records. Specifically, i2b2 has provided sets of fully deidentified notes and proposed several challenges, such as deidentification and heart disease risk factor challenge, temporal relation challenge, conference challenge, relation challenge, medication challenge, obesity challenge, and deidentification and smoking challenge.

In addition, the TREC Medical Records Track (TREC2011-2012) [4] was established for the research community to focus on the problem of providing content-based access to free text fields of electronic health records. Then, Clinical Decision Support/Precision Medicine Tracks (TREC2014-2018) [5-9] were organized in TREC. The Clinical Decision Support Track focused on the retrieval of biomedical articles relevant for answering generic clinical questions about medical records, and TREC Precision Medicine Track focused on a use case in clinical decision support, providing useful precision medicine-related information to clinicians treating cancer patients.

Furthermore, the CLEF eHealth [10] focused on NLP and information retrieval for clinical care in the European Union. In Japan, NTCIR Medical tasks and MedNLP workshops (MedNLP-1, MedNLP-2, and MedNLP-Doc) [11-16] were organized to promote and support the generation of practical tools and systems applicable in the medical industry, which will support medical decisions and treatments by physicians and medical staff. MedNLP-1 [11,14] aimed to retrieve important information (personal and medical) from the clinical text written in Japanese. MedNLP-2 [12,15] challenged to extract information from medical reports written by physicians and from past medical exams. MedNLP-Doc [13,16] proposed a task to guess the name of the disease (represented by the International Codes for Diseases [ICD]) from the provided medical records. However, to the best of our knowledge, the MedWeb is the first shared task for dealing with health-related social media data.

Due to the widespread use of the internet, considerable material concerning medical care or health has been made available on the Web, especially social media such as Twitter and Facebook. Furthermore, various Web mining techniques for utilizing the material have been developed. One of the most popular medical applications is disease surveillance, which aims to predict disease epidemics based on the use of disease-related terms. Particularly, influenza surveillance using social media has been extensively studied [17-27,55]. As most previous studies have relied on shallow textual clues in messages, such as the number of occurrences of specific keywords (eg, *flu* or *influenza*), there are several noisy messages. To filter out noisy tweets, a binary classifier has been employed. In contrast, the MedWeb has challenged a more difficult and practical task of performing a multi-label classification of cross-language user-generated messages.

### Conclusions

This paper provided an overview of the NTCIR-13 MedWeb task, which was designed as a more generalized task for public surveillance, focusing on social media (such as Twitter). In particular, the task’s goal was to classify symptom-related messages. This task had 2 characteristics: (1) multi-label (cold, cough, diarrhea, fever, hay fever, headache, flu, and runny nose) and (2) cross-language (Japanese, English, and Chinese). The results empirically demonstrated that an ensemble of multiple machine learning methods was effective in terms of classification of cross-language messages with multiple labels. We believe that the findings would be a foundation for future and deeper approaches for disease surveillance with social media data.

## Appendix

Multimedia Appendix 1 Organization of groups participating in MedWeb and statistics of result submissions. Note that it is listed in alphabetical order by Group ID, and ja, en, and zh correspond to Japanese, English, and Chinese subtasks, respectively.

## Abbreviations

CLEF: Cross-Language Evaluation Forum for European Languages
CNN: convolutional neural network
i2b2: Informatics for Integrating Biology and the Bedside
ICD: International Codes for Diseases
MedNLP: Medical Natural Language Processing
MedWeb: Medical natural language processing for Web document
NLP: natural language processing
NTCIR: NII Testbeds and Community for Information access Research
SVM: support vector machine
TREC: Text Retrieval Conference
