# Supplementary material for: Tweet Classification Toward Twitter-Based Disease Surveillance: New Data, Methods, and Evaluations
Source: J Med Internet Res. 2019 Feb 20;21(2):e12783. doi: 10.2196/12783 (PMC6401666; doi:10.2196/12783)
Supplement: Multimedia Appendix 1 [file jmir_v21i2e12783_app1.pdf]

Multimedia Appendix 1

Organization of groups participating in MedWeb and statistics of result submissions. Note that It is listed in alphabetical order by Group ID and ja, en, and zh mean Japanese, English, and Chinese subtasks, respectively.

Group ID	Organization	Type	ja	en	zh
AITOK [32]	Tokushima University, Japan	Academia	2		
AKBL [33]	Toyohashi University of Technology, Japan	Academia	3	3	
DrG [34]	The University of Tokyo, Japan	Academia	1		
KIS [35]	Shizuoka University, Japan	Academia	3		
NAIST [36]	Nara Institute of Science and Technology, Japan	Academia	3	3	3
NIL [37]	NIL Software Corp., Japan	Industry	1		
NTTMU [38]	Taipei Medical University, Taiwan	Academia	3	3	
TUA1 [39]	Tokushima University, Japan	Academia			3
UE [40]	University of Evora, Portugal and Nikon Corp., Japan	Joint (Academia and Industry)	3	3	
